# Efficacy of parenteral vaccination against tuberculosis with heat-inactivated *Mycobacterium bovis* in experimentally challenged goats

**DOI:** 10.1371/journal.pone.0196948

**Published:** 2018-05-09

**Authors:** Claudia Arrieta-Villegas, Tania Perálvarez, Enric Vidal, Zoë Puighibet, Xavier Moll, Albert Canturri, Iker A. Sevilla, Yvonne Espada, Ramón A. Juste, Mariano Domingo, Bernat Pérez de Val

**Affiliations:** 1 IRTA, Centre de Recerca en Sanitat Animal (CReSA, IRTA-UAB), Campus UAB, Bellaterra, Barcelona, Catalonia, Spain; 2 Departament de Medicina i Cirurgia Animals, Universitat Autònoma de Barcelona (UAB), Bellaterra, Barcelona, Catalonia, Spain; 3 Departament de Sanitat i Anatomia Animals, Universitat Autònoma de Barcelona (UAB), Bellaterra, Barcelona, Catalonia, Spain; 4 NEIKER-Tecnalia, Instituto Vasco de Investigación y Desarrollo Agrario, Departamento de Sanidad Animal, Derio, Bizkaia, Basque Country, Spain; 5 SERIDA, Servicio Regional de Investigación y Desarrollo Agroalimentario, Centro de Biotecnología Animal, Gijón, Asturias, Spain; Public Health England, UNITED KINGDOM

## Abstract

Tuberculosis (TB) in animals is a re-emerging disease with a wide range of hosts that causes large economic losses in livestock. Goats are particularly susceptible to TB and, in endemic areas, vaccination may be a valuable measure to control the disease. The main aim of this study was to evaluate the efficacy of parenteral vaccination of goats with a heat-inactivated *Mycobacterium bovis* (HIMB) vaccine, and compare it to M. *bovis* Bacille Calmette–Guérin (BCG) vaccine. Twenty-four goat kids were divided in 3 groups as following: HIMB vaccinated group (n = 8), BCG vaccinated group (n = 8) and unvaccinated group (n = 8). Afterwards, goats were experimentally challenged with *Mycobacterium caprae* by the endobronchial route. Antigen specific interferon-γ release assays and serology were performed after vaccination and challenge. Pathological and bacteriological parameters were evaluated after necropsy at 9 weeks post-challenge (p.c.). HIMB vaccine showed similar levels of protection to BCG in terms of volume reduction of thoracic TB lesions, presence of extra-pulmonary lesions, as well as a slight reduction of bacterial load in pulmonary lymph nodes. Moreover, HIMB vaccine did not induce interferences on the interferon-γ release assay based on reagents previously developed to differentiate infected from BCG vaccinated individuals. The results indicate that HIMB is a suitable vaccine candidate for further larger-scale trials under field conditions in goats.

## Introduction

Animal tuberculosis (TB) is a re-emerging multi-host disease caused by microorganisms belonging to the *Mycobacterium tuberculosis* complex (MTBC), such as *Mycobacterium bovis* and *Mycobacterium caprae*, that may affect a wide range of domestic animals and wildlife and poses a risk of infection for humans [1]. TB in livestock causes economic losses in agricultural industries [2]. Goats are natural hosts of both *M*. *caprae* and *M*. *bovis*, and can be a source of TB infection for other epidemiologically related species such as cattle [3] or sheep [4]. Thus the lack of an official TB control program in goats can jeopardize the eradication efforts in cattle [5].

In this scenario, vaccination of goats may be a useful long-term tool to reduce TB prevalence in goat herds. *M*. *bovis* Bacillus Calmette-Guérin (BCG) is the only vaccine licensed for humans and it has also been also licensed for badgers [6]. BCG efficacy has been evaluated in different experimental animal models with heterogeneous results [7]. In experimentally challenged goats, BCG afforded protection by reduction of pulmonary disease severity and preventing extra-pulmonary dissemination [8,9]. Finally, as a live-attenuated vaccine, BCG stability in environmental conditions could be limited and an eventual transmission to non-vaccinated animals cannot be excluded [10].

The Heat-Inactivated *Mycobacterium bovis* (HIMB), is a new vaccine candidate that may rule out some constraints of live-attenuated vaccines [11]. The efficacy of HIMB has already been evaluated under experimental conditions in cattle [12], sheep [13], red deer [14] and wild boar [15] and, under field conditions in wild boar [16], yielding variable results.

The aim of this study was to evaluate the efficacy of parenteral HIMB vaccination (injected i.m. and s.c) in comparison with parenteral BCG vaccination in *M*. *caprae* experimentally challenged goats, by studying cell-mediated and humoral immune responses after vaccination and challenge, and TB lesion volume reduction. Additionally, the effects of vaccination on IFN-γ release assay (IGRA) based TB diagnostic were evaluated.

## Materials and methods

### Animals and experimental design

Twenty-four *Murciano-granadina* goat kids, aged from 4 to 7 weeks and acquired from a farm with no history of TB, were selected based on negative results to IGRA (ID Screen^®^ Ruminant IFN-γ, ID.vet, Grabels, France) and all animals were accommodated in a pen of an experimental farm. Animals were randomly distributed in 3 experimental groups of 4 males and 4 females each. Then, distribution was corrected by weight, in order to have homogenous characteristics in each group. One group of 8 animals was subcutaneously vaccinated with a live attenuated *M*. *bovis* BCG vaccine (BCG group), another group of 8 animals (HIMB group) was parenterally vaccinated with the heat-inactivated *M*. *bovis* vaccine (4 were vaccinated subcutaneously and 4 intramuscularly with the purpose to investigate adverse reactions) and, finally, the last 8 animals remained unvaccinated (Control group). At seven weeks post vaccination (p.v.) goat kids were placed in biosafety level (BSL) 3 containment facilities at IRTA-CReSA, in two boxes, one for unvaccinated animals and another for both BCG and HIMB vaccinated animals. Finally, one week later (8 weeks p.v.), the experimental challenge with *M*. *caprae* was performed.

Goat kids were vaccinated at week 0. Blood samples were taken from jugular vein in heparinized blood tubes at weeks 0, 3, 5, 8, 11, 13, 15 and 17 of the experiment. Clinical signs of TB were monitored after the experimental infection and animals were weighed at weeks 0, 8, 11, 13, 15 and 17. Finally, rectal temperature was measured weekly p.c.

### Ethics statement

All animal procedures used during this experiment were approved by the Animal Welfare Committee of the Universitat Autònoma de Barcelona and the Generalitat de Catalunya (Procedure Number 8697), and in conformity with European Union Laws for protection of experimental animals (2010/63/EU).

### *M*. *bovis* BCG vaccine

*M*. *bovis* BCG Danish 1331 strain (ATCC, Ref.35733^™^) vaccine stock was prepared as described previously [9]. Then, BCG was diluted in sterile Phosphate Buffered Saline (PBS) to reach a suspension of 10^6^ colony forming units (CFU)/ml, and 0.5 ml of the suspension (5 × 10^5^ CFU) was subcutaneously inoculated at the right axilla.

### Heat-inactivated *M*. *bovis* vaccine (HIMB)

The *M*. *bovis* strain (SB0339) used was first isolated from a naturally infected wild boar on Coletsos medium. The vaccine was prepared as described by Balseiro *et al*. [13]. The inactivated *M*. *bovis* suspension was adjuvated with Montanide^™^ ISA 50V2 (Seppic, Paris, France) to form a water in oil emulsion in a proportion 1:1 and contained approximately 10^7^ CFU of heat-treated bacteria per dose (1 ml). Animals were injected subcutaneously, at the right axilla, or intramuscularly, at the right semitendinosus muscle.

### *M*. *caprae* inoculum preparation and experimental challenge

The field strain *M*. *caprae* SB0416 (www.Mbovis.org*)* used for the inoculum was subcultured in Middlebrook 7H9 medium and titrated in 7H11 plates (BD diagnostics, Sparks, USA) as described previously [9]. For challenge, an aliquot was used for preparing the inoculum by diluting it with sterile PBS to attain a final suspension of 2 × 10^4^ CFU/ml of *M*. *caprae*.

Goat kids were premedicated by an intramuscular injection with a cocktail of 0.05 mg/kg of acepromazine maleate (Equipromacina^®^) and 0.2 mg/kg of butorphanol tartrate (Torbugesic^®^). After sedation, they were intravenously anesthetized with propofol (Propofol Lipuro^®^) at 4–6 mg/kg and midazolam (Dormicum^®^) at 0.2mg/kg. Anesthetized animals were endobronchially challenged with a 0.5 ml of *M*. *caprae* inoculum (each animal received 10^4^ CFU) as previously described [17].

### Whole-blood IFN-γ release assay (IGRA)

Blood samples were collected at weeks 0, 3, 5, 8, 11, 13, 15 and 17, and were processed as described by Pérez de Val et al., [18]. Shortly, 3 aliquots of 900 μl of whole blood were added into 3 wells of 96-well cell culture plates (Eppendorf Ibérica, Madrid, Spain), two wells were subsequently stimulated with *M*. *bovis* (PPD-B) and *M*. *avium* (PPD-A) tuberculins (CZ Veterinaria, Porriño, Galicia, Spain), both at a final concentration of 20 μg/ml and PBS (Sigma-Aldrich, Steinheim, Germany) was added in the other well as the unstimulated control. In addition, 225 μl of whole blood were stimulated with a mixture of ESAT-6 and CFP-10 (EC) recombinant proteins (Lionex, Braunschweig, Germany), used at a final concentration of 10 μg/ml each. Samples were incubated at 37 °C with CO_2_ overnight. Finally, plasma was collected and analyzed by ruminant IFN-γ ELISA (ID.vet) following the manufacturer instructions. ELISA was read at 450 nm using a spectrophotometer (Biotek Power Wave XS). The interpretation of tuberculin-based IGRA results was performed according the two cut-off points of sample-to-positive ratios (S/P) recommended by the manufacturer, i.e. ((Optical density (OD) of PPD-B–OD of PPD-A) / (OD mean kit positive control (CP)–OD mean kit negative control (CN))) × 100. A sample was considered as positive if S/P ≥ 35% (conservative criterion) or ≥ 16% (stringent criterion). In addition, EC-specific IGRA results were calculated as following: S/P = ((OD of EC–OD of PBS) / (OD CP–OD CN)) × 100. A sample was considered as positive if S/P ≥ 16%.

### Serology

Plasma samples were analyzed in duplicate for antibodies against the cell-surface lipoprotein MPB83 (Lionex), specific for *M*. *tuberculosis* Complex (MTBC), using a homemade ELISA as described previously [19]. A sample was classified as positive when the ΔOD 450 nm (sample wells average OD_450 nm_ minus the blank well OD _450nm_) was equal or higher than 0.2 (the optimal cut-off point previously determined by Pérez de Val et al. [19].

### Post-mortem examination

Goats were euthanized at week 17 (week 9 p.c.) by intravenous injection of a sodium pentobarbital overdose. A complete necropsy procedure was conducted, pulmonary (tracheobronchial, mediastinal cranial and caudal) lymph nodes (LN) were carefully removed and sliced, and then the diameters of each lesion were measured. The approximate volume of gross lesions was calculated using the formula of the most similar geometrical morphology of each lesion (sphere, cylinder or prism). After slicing, whole pulmonary LN were frozen and stored for later bacterial culture. All remaining viscera were also examined and other extra-pulmonary tissues with presence of TB-like lesions were collected and subsequently fixed in 10% buffered formalin for histopathological confirmation by Hematoxylin/Eosin staining. Finally, the whole lungs were filled with formalin as previously described [17] and one month later, 20 lungs (6 from the control group, and 7 from each vaccinated group) were analyzed by computed tomography (CT).

### Computed tomography (CT)

After fixation, the extension of the pathology in lungs was assessed by 16-slice multi-detector CT scanner (Brivo CT-385, GE Healthcare, UK) as previously described [13]. Briefly, volume rendering (VR) was employed to calculate the whole volume of each lung. Different density patterns (calcified lesions, cavitary lesions and solid lesions) were used to settle down tuberculous lesions in lungs, and to determine its volume by 2D, 3D images and VR, using multiplanar reconstructions. Calcified lesions were selected by their Hounsfield units (range 80–300 HU) and the total volume of them was calculated.

### Bacterial culture and count

Whole pulmonary LN of each animal were thawed, pooled, homogenized and decontaminated as previously described [17]. Four ten-fold serial dilutions of tissue homogenates in sterile PBS were performed and 100μl of each dilution were plated on Middlebrook 7H11 medium (Ref.: I01S01687820, BD diagnostics). All the cultured plates were incubated at 37 °C for 28 days. Finally, CFU were counted and the total bacterial burden in LN of each animal was estimated. MTBC colonies were confirmed by multiplex PCR, as described by Wilton et al. [20].

### Data analysis

Non parametrical Kruskal Wallis test, followed by pair-wise comparisons with the non-parametric one-tailed Wilcoxon rank sum test with Bonferroni correction was used to assess differences among groups in mean rectal temperature, weight increase, bacterial load (log_10_ CFU transformed counts) and pathological variables. For antigen-specific IFN-γ responses and MPB83-IgG responses, the same statistical tests were performed with two-tailed significance. Differences in the frequency of extra-pulmonary TB lesions among groups were assessed using a Fisher exact test. Statistical significance was established when *P*-value < 0.05. Statistical analysis was performed with Deducer for R package V2.15.0 (R Foundation for Statistical Computing, Vienna, Austria).

## Results

### Clinical signs and body condition

No adverse reactions to vaccinations were observed at the site of vaccine injection. Neither clinical signs nor remarkable changes in body conditions were observed after vaccination and prior to challenge. After challenge, one animal of HIMB group did not recover from anesthesia. Clinical signs appeared in some animals at 5 weeks p.c. and all unvaccinated animals showed clinical signs at the end of the experiment. The clinical sings mostly observed were cough and dyspnea, while some animals showed anorexia and/or lethargy at the last time point. Table 1 shows the proportion of animals with clinical signs recorded after challenge. A goat from the control group with dyspnea, anorexia and lethargy was euthanized for ethical reasons at week 16.

**Table 1:** Number of animals with clinical signs after *M*. *caprae* challenge.

Group	Week				
	8	11	13	15	17
Control	0/8	0/8	5/8	6/8	8/8^a^
BCG	0/8	0/8	4/8	4/8	4/8
HIMB	0/7	0/7	2/7	6/7	4/7^b^

Animals were challenged at week 8. All animals with clinical signs showed cough and dyspnea.

^a^ One goat was anorexic, another lethargic and one with both signs was euthanized at week 16.

^b^ Two goats were anorexic.

**Fig 1:**
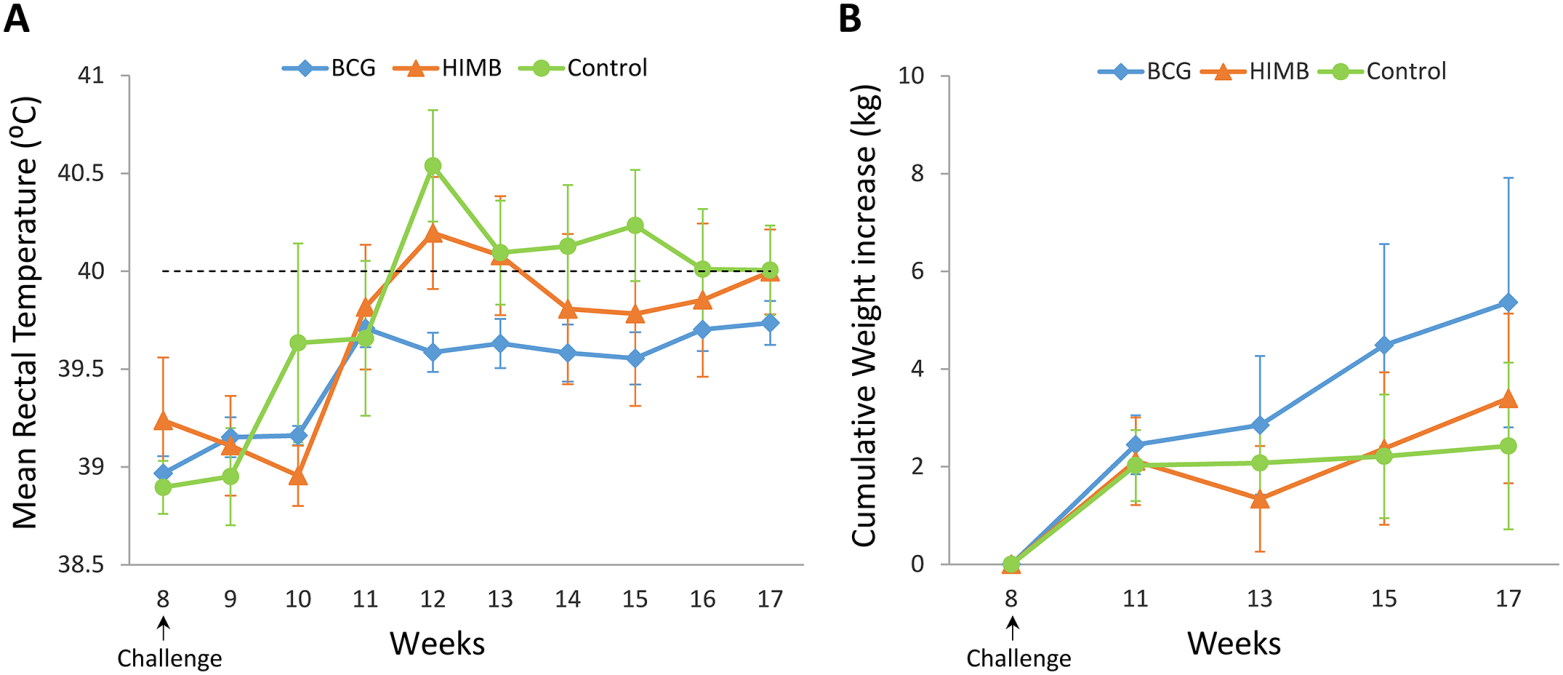
Clinical signs after *M*. *caprae* challenge. (**A**) Rectal temperature. Results are expressed as mean rectal temperature (°C) ± 95% confidence interval (CI). Horizontal dashed line shows the threshold used for defining fever (40 °C). (**B**) Body weight increase. Results expressed as increase of weight (kg) ± 95% CI from the week of challenge. Groups: control (n = 8), BCG vaccinated (n = 8) and HIMB vaccinated (n = 7) animals.

A peak of mean rectal temperature (above 40 °C) was detected at week 12 (4 weeks p.c.) in control and HIMB groups when compared to BCG group (*P* = 0.007 and *P* = 0.031, respectively). In control group, the mean rectal temperatures remained above 40 °C from week 12 until the end of the experiment, whereas mean rectal temperatures in HIMB group showed a mild decrease after week 12. BCG group showed the lowest mean rectal temperatures throughout the experiment, reaching the maximal mean rectal temperature at week 11, without attaining 40 °C (See Fig 1A).

The mean body weight increase for each group after challenge is shown in Fig 1B. BCG vaccinated group showed weight gain in all time points after challenge, however differences were not statistically significant when compared to the other groups. Unvaccinated group did not show weight gain from 3 weeks p.c. onwards, while HIMB vaccinated group started to show weight gain after week 13 (5 weeks p.c.).

### Interferon-γ responses after vaccination and challenge

**Fig 2:**
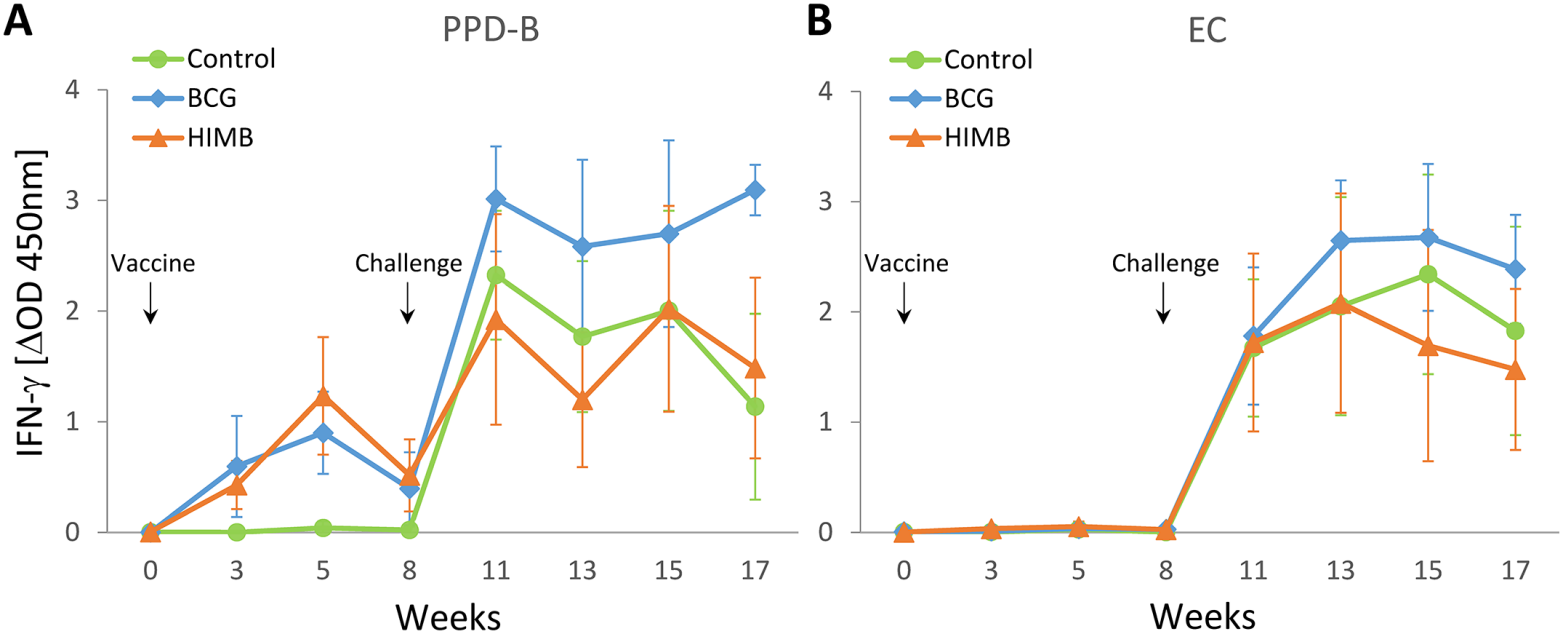
Antigen specific IFN-γ responses after vaccination and *M*. *caprae* challenge. The graphic shows the levels of IFN-γ measured by ELISA. Results are expressed as ΔOD_450mn_ ± 95% CI. (**A**) Response against Bovine tuberculin (PPD-B). (**B**) Response against EC antigen cocktail (ESAT-6 / CFP-10). Groups: Control (n = 8), BCG (n = 8), HIMB (n = 7).

The mean IFN-γ responses before and after challenge for each treatment group are represented in Fig 2. The IFN-γ response to PPD-B (Fig 2A) started to increase after vaccination in both vaccinated groups when compared to control group from week 3 to 8 (at week 3, *P* = 0.001 for both vaccinated groups; at week 5, *P* = 0.004 and *P* = 0.035; and at week 8, *P* = 0.066 and *P* = 0.008, for BCG and HIMB group, respectively). All BCG vaccinated and 7 out of 8 HIMB vaccinated (3 s.c. and 4 i.m.) animals showed detectable PPD-B-specific IFN-γ responses after vaccination. The other subcutaneously HIMB vaccinated animal did not show any detectable response from vaccination to *M*. *caprae* challenge (data in S1 Table). Three weeks after challenge (week 11), all animals showed high IFN-γ responses to PPD-B, although they were slightly higher in the BCG group, being statistically significant when compared to HIMB group at week 13 (*P* = 0.044) and to both control and HIMB groups at week 17 (*P* = 0.031 and *P* = 0.044, respectively, Fig 2A). After vaccination, IFN-γ to EC was undetectable among groups, but after challenge, all animals responded to EC, although no differences were observed (Fig 2B).

Qualitative results of the IGRA to both PPD-B and EC are shown in Table 2. After vaccination, in both vaccinated groups positive animals to PPD-B were detected (positivity varied according to the criterion used) and no positive animals to EC were observed regardless of treatment group. All animals showed positivity to both reagents p.c., except at the last week when some animals from control group were negative to PPD-B, especially when the conservative criterion was used.

**Table 2:** Number of positive goats to each IFN-γ release assay.

**Antigen**	**Group**	**Week**							
		**0**		**3**		**5**		**8**	
		C	S	C	S	C	S	C	S
*PPD-B*^b^	Control	0/8	0/8	0/8	0/8	0/8	0/8	0/8	0/8
	BCG	0/8	0/8	2/8	5/8	4/8	6/8	1/8	5/8
	HIMB	0/8	0/8	1/8	2/8	4/8	4/8	3/8	3/8
*EC*^c^	Control	0/8		0/8		0/8		0/8	
	BCG	0/8		0/8		0/8		0/8	
	HIMB	0/8		0/8		0/8		0/8	
		**Week**							
		**11**		**13**		**15**		**17**^a^	
		C	S	C	S	C	S	C	S
*PPD-B*^b^	Control	8/8	8/8	8/8	8/8	8/8	8/8	5/7	6/7
	BCG	8/8	8/8	8/8	8/8	8/8	8/8	8/8	8/8
	HIMB	6/7	6/7	7/7	7/7	6/7	7/7	7/7	7/7
*EC*^c^	Control	8/8		8/8		8/8		8/7	
	BCG	8/8		8/8		8/8		8/8	
	HIMB	7/7		7/7		6/7		7/7	

Animals were vaccinated at week 0 and challenged at week 8.

^a^ One goat of the Control group was euthanized at week 16.

^b^ PPD-B, *M*. *bovis* purified protein derivative; two criteria were used for positivity: C, Conservative (S/P ≥ 35%), and S, Stringent (S/P ≥ 16%).

^c^ EC, ESAT-6/CFP-10 protein mixture; the criterion for positivity was S/P ≥ 16%.

### Humoral responses after vaccination and challenge

**Fig 3:**
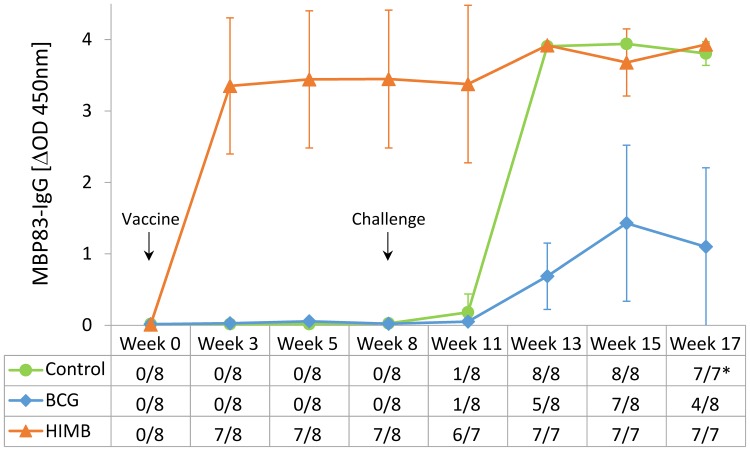
Antibody responses to MPB83 after vaccination and challenge. The graphic shows the MPB83-IgG levels measured by ELISA. Results are expressed as ΔOD_450mn_ ± 95% CI. Groups: Control (n = 8), BCG (n = 8), HIMB (n = 7). The table in the horizontal axis represents the qualitative results of the test (No. of seropositive goats/total goats).*One goat of the control group was euthanized at week 16.

The mean MPB83-specific IgG levels (ΔOD) measured by ELISA, before and after challenge, and qualitative results of serology are shown in Fig 3. Three weeks after vaccination, mean IgG levels of MPB83 dramatically increased in HIMB vaccinated group (at week 3, *P* = 0.021 when compared to both BCG and control group; at week 5, *P* = 0.030 and *P* = 0.008; and at week 8, *P* = 0.021 and *P* = 0.031, when compared to BCG and control group, respectively). Nonetheless, one subcutaneously HIMB vaccinated animal did not show any detectable serological response to MPB83 after vaccination, while the rest of HIMB vaccinated animals showed strong responses at a similar level (data in S2 Table). Five weeks after challenge (week 13), all animals presented serological responses against *M*. *caprae*. In control group, levels of MPB83-IgG sharply raised and in HIMB group, a slight boost of serological response was observed. However, the BCG group showed the lowest MPB83-IgG levels p.c. compared to both control and HIMB groups (at week 13, *P* = 0.003 and *P* < 0.001, at week 15 *P* = 0.004 and *P* = 0.018, and at week 17, *P* = 0.007 and *P* = 0.006, when compared to control and HIMB groups, respectively).

With regard to qualitative analysis of humoral response to MPB83, HIMB vaccinated animals exhibited positive results after vaccination, except for one animal. In contrast, control and BCG group did not show any positive animal before challenge. Five weeks after challenge, in control and HIMB groups all animals were positive and remained so until the end of the experiment. On the other hand, in BCG group the number of positive animals fluctuated during the p.c. period.

### Post mortem findings

**Fig 4:**
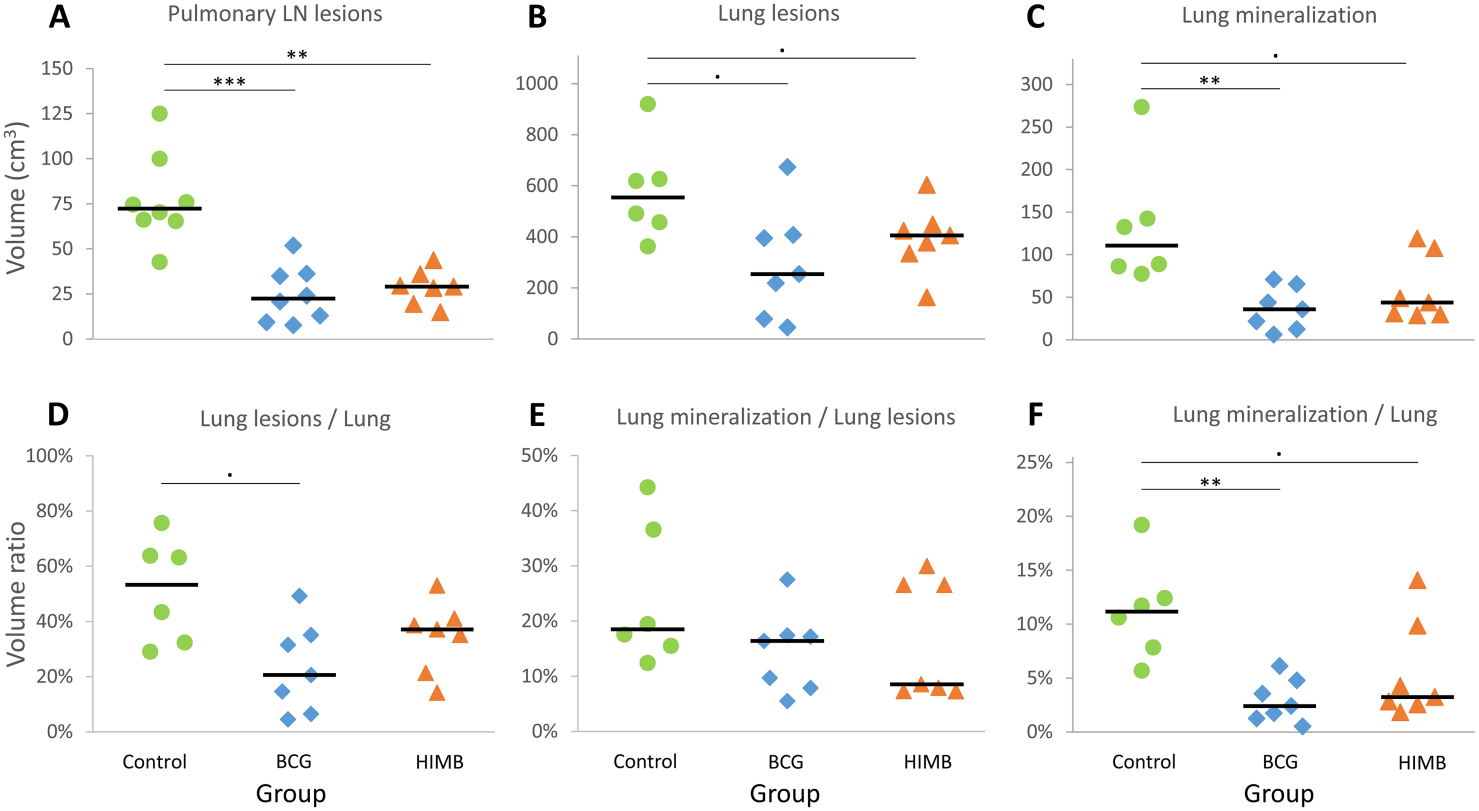
Quantitative pathological results. (**A-C**) Individual volumes of TB lesions expressed in cm^3^. (**D-F**) Individual ratios between volumes expressed in %. (**A**) Total volume of lesions in pulmonary lymph nodes (LN). Groups: Control (n = 8), BCG (n = 8) and HIMB (n = 7). (**B**) Total volume of lung lesions. (**C**) Total volume of mineralized lesions in lungs. (**D**) Ratio of total volume of lung lesions / volume of the whole lung. (**E**) Ratio of total volume of lung mineralization / total volume of lung lesions. (**F**) Ratio of total volume of lung mineralization / volume of the whole lung. Groups (**B-F**): Control (n = 6), BCG (n = 7) and HIMB (n = 7). Horizontal lines represent median values. · *P* < 0.1, * *P* < 0.05, ** *P* < 0.01, *** *P* < 0.001, Kruskal-Wallis test with the *post hoc* Wilcoxon rank sum test with Bonferroni correction.

All goats presented extensive lung TB lesions at necropsy. The assessment of TB lesions and pathological parameters are shown in Fig 4. When compared to the control group, volume of pulmonary LN lesions in BCG and HIMB vaccinated groups were significantly lower (*P* < 0.001 and *P* = 0.002, respectively), volume of lung lesions were also lower, but yet not statistically significant (*P* = 0.075), with also lower volume of mineralization in lungs (*P* = 0.002 and *P* = 0.052, respectively). However, when the ratio lesion volume / lung volume was assessed, only BCG group showed a slightly lower ratio than the control group (*P* = 0.05). Regarding the mineralization volume / lesion volume ratio, no differences were observed among groups. However, both BCG and HIMB vaccinated groups showed lower mineralization volume / lung volume ratios than the control group (*P =* 0.004 and *P =* 0.079, respectively). For the pathological parameters described above, no statistical differences were detected between vaccinated groups.

The total volume of lesions in lungs and pulmonary LN in the BCG group (median: 290 cm^3^, IQR: 411–178) and in HIMB group (median: 449 cm^3^, IQR: 468–377) were significantly lower than in the control group (median: 625 cm^3^, IQR: 734–537, *P* = 0.024, *P* = 0.042; respectively), whereas no significant differences were observed between vaccinated groups (*P* = 0.778).

TB lesions were predominant in lungs and pulmonary LN in the three groups. However, the number of animals with extra-pulmonary lesions was significantly lower in both BCG and HIMB vaccinated groups, 1/8 and 2/7, respectively, while unvaccinated animals showed 7/8 animals with extra-pulmonary lesions (*P* = 0.005 and *P* = 0.024 respectively; Table 3).

**Table 3:** Distribution of extra-pulmonary lesions among groups.

Group	No. of animals with extra-pulmonary lesions	RF LN	MS LN	Liver	Spleen	GS LN	RH LN
Control	7/8	1/8	5/8	1/8	2/8	1/8	1/8
BCG	1/8**	0/8	0/8	0/8	1/8	0/8	0/8
HIMB	2/7*	1/7	1/7	0/7	0/7	0/7	0/7

LN: Lymph node. RF: Retropharyngeal LN (not confirmed by histopathology). MS: Mesenteric LN. GS: Gastrosplenic LN, RH: Retro-Hepatic LN.

* *P* < 0.05,

** *P* < 0.01, Fisher exact test.

**Fig 5:**
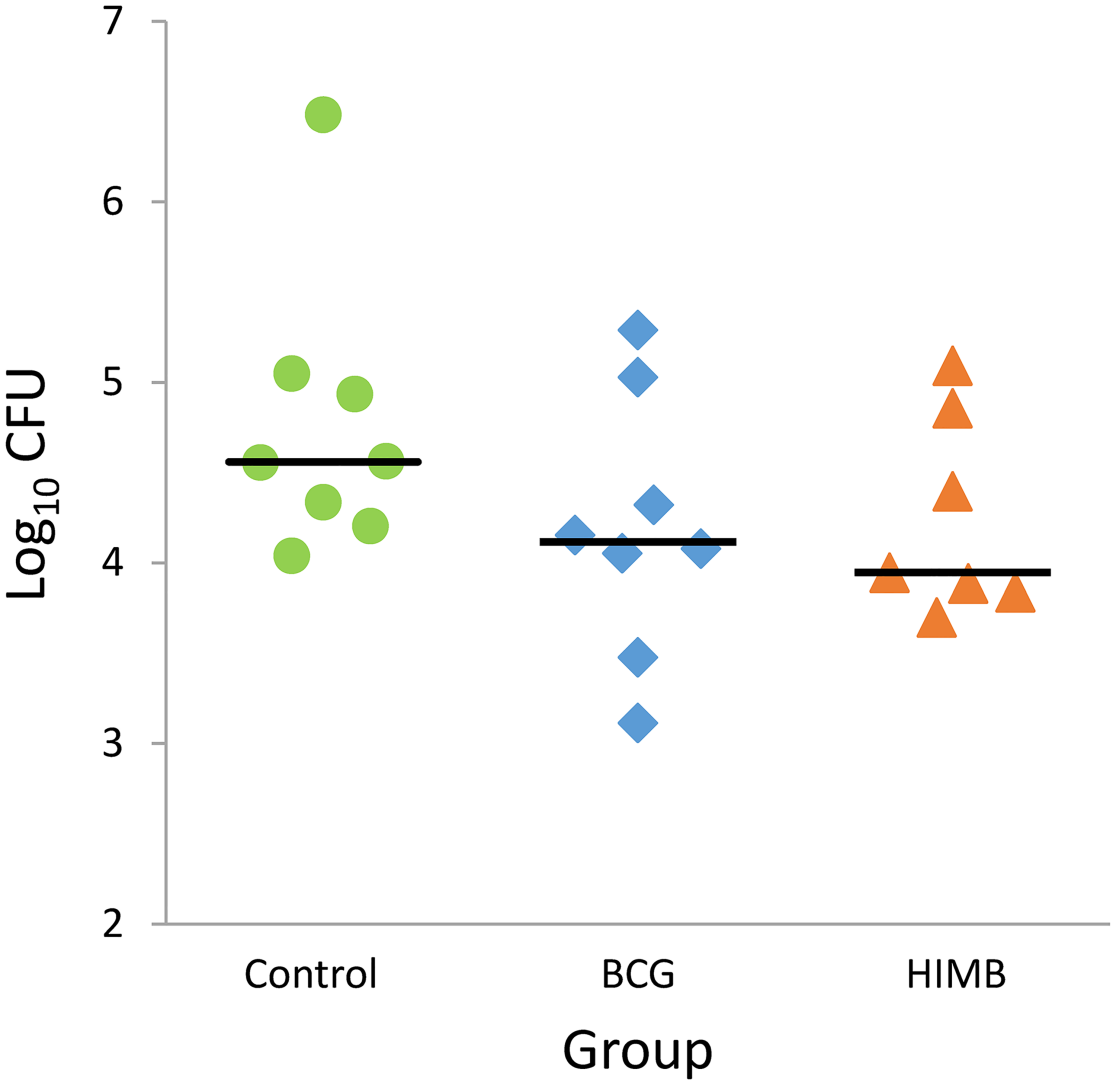
Bacterial load in pulmonary lymph nodes. Results are expressed as log_10_ CFU for each group. Groups: Control (n = 8), BCG (n = 8) and HIMB (n = 7). Horizontal lines represent median values.

The bacterial load in pulmonary LN in the unvaccinated group (median: 4.56 log_10_ CFU, IQR: 4.96–4.30) was slightly higher than in BCG and HIMB vaccinated groups (median: 4.12 log_10_ CFU, IQR: 4.50–3.91, and median: 3.95 log_10_ CFU, IQR: 4.63–3.86) but no statistical differences were detected among groups (Fig 5).

## Discussion

In this study, the efficacy of a heat inactivated *M*. *bovis* vaccine in a goat model was evaluated in comparison to BCG vaccinated and control unvaccinated goats. The results indicated that parenteral HIMB vaccination of goats confers protection, mainly in terms of volume lesions reduction in both lungs and pulmonary LN, being comparable with BCG vaccinated animals. In accordance to these results, previous studies showed similar degree of protection in orally and parenterally vaccinated wild boar [15], using a slightly different HIMB inactivation procedure (80 °C for 30 min. instead of 83–85 °C for 45 min.), and in orally vaccinated red deer [14], using the same HIMB inactivation procedure than in the present study. On the contrary, HIMB vaccination through the oral route did not show protection in *M*. *caprae* experimentally challenged lambs [13].

Even though all goats showed TB lesions in the thoracic cavity irrespectively of the treatment group, BCG and HIMB vaccinated groups showed a significant reduction of mean volume of TB lesions in lungs and in pulmonary LN. These results are in accordance to those previously observed in HIMB vaccinated red deer [14] and wild boar [15], that showed a reduction in the percentage of lung lobe affectation compared to control group. Furthermore, a significant reduction of the presence of TB lesions in extra-pulmonary tissues was observed in both vaccinated groups when compared to the unvaccinated group. These results are consistent with previous BCG vaccination studies in goats, under experimental [8] and field conditions [21]. In contrast, HIMB orally vaccinated but not BCG orally vaccinated red deer showed a reduction of presence of extra-pulmonary lesions [14].

Besides, no adverse reactions at the inoculation point (in either subcutaneous or intramuscular delivery) were observed after HIMB vaccination, as previously reported in parenterally vaccinated wild boar [11,16]. As expected, no adverse reactions were observed after subcutaneous vaccination of goats with BCG, in consistency with those previously reported in a BCG safety study in goats [18].

The lower *M*. *caprae* burden in pulmonary LN of vaccinated groups compared to unvaccinated animals, suggests a reducing effect of vaccines in the mycobacterial drainage from lungs to pulmonary LN. Previous studies indeed demonstrated a significant reduction of bacterial load in lungs and pulmonary LN in parenterally BCG vaccinated badgers [22] and goats [8,9], respectively. In fact, these badgers were challenged endobronchially with a similar dose than the one in the present study (~10^4^ CFU), but goats were challenged with a lower dose of *M*. *caprae* (10^3^ CFU). In contrast, in a high dose *M*. *bovis* (10^6^ CFU) challenge conducted in red deer using the intratracheal route [14], no differences in bacterial load in pulmonary LN were observed among groups (HIMB and BCG orally vaccinated animals and unvaccinated animals). All these findings suggest that the challenging dose may influence the bacterial burden found in LN, thus interacting with the vaccine effect.

In HIMB vaccinated and unvaccinated goats, a peak of fever was registered at 4 weeks p.c. and was consistent with a weight gain cessation observed in both groups one week after. Similar results were described in HIMB orally vaccinated lambs [13]. On the other hand, BCG vaccinated animals did not show fever neither weight losses, as previously described in BCG vaccinated goats [9] and lambs [13].

In the present study, HIMB and BCG vaccines interfered on the diagnosis of TB using the IGRA with standard tuberculins. Thus, some vaccinated animals (2/8 and 1/8, from BCG and HIMB vaccinated animals, respectively) were positive to the tuberculin-based IGRA after vaccination, when assessed with the conservative criterion. Positivity increased when the stringent criterion was used, mainly in BCG group (5/8) and, in lower number of cases, in the HIMB group (2/8). IFN-γ responses against tuberculins were previously observed after parenterally HIMB vaccination in goats [23], cattle [12,24], and wild boar [15].

As expected, EC-specific IFN-γ responses were not detected after BCG vaccination as previously described in goats [8,9,18]. The BCG genome does not contain the genes codifying for ESAT-6 and CFP-10, but these genomic region is not deleted in the virulent *M*. *bovis* strain from which HIMB vaccine was originally obtained. Interestingly, no detectable IFN-γ responses to EC protein mixture were observed after HIMB vaccination, as previously described in orally vaccinated lambs [13] and cattle [24]. On the contrary, parenterally HIMB vaccinated cattle were positive to EC protein mixture and EC peptide cocktail-based IGRAs [24]. In another study, no detectable responses to single ESAT-6 protein were detected in cows subcutaneously vaccinated with HIMB, whereas, slight responses were observed when single CFP-10 protein was used [12]. However, in other studies, experimentally challenged goats showed low IFN-γ responses to ESAT-6 protein compared to EC peptide cocktail [8], and experimentally challenged lambs showed similar IFN-γ responses to both EC protein mixture and peptide cocktail [13]. This lead to speculate that these antigens may be present in HIMB vaccine but at an undetectable concentration, or are present in an altered form induced by the vaccine inactivation [12]. The results of the present study suggest that the EC protein mixture might be the most useful DIVA reagent in HIMB vaccinated goats, although further longer and larger-scale studies are required to confirm these results.

With regard to the antibody dynamic, HIMB vaccinated animals showed a rapid and strong seroconversion against MPB83 after vaccination (3 weeks p.v.). Similar results were previously found in parenterally HIMB vaccinated wild boar [15] and cattle [12]. By contrast, MTBC-specific antibody responses were not detected after oral vaccination of lambs [13] and wild boar [15]. After challenge, HIMB vaccinated animals remained with high levels of MPB83-IgG, reaching the saturation levels of the test, thus, the effect of challenge on serological responses could not be evaluated in this group. Interestingly, the control group showed a rapid and strong seroconversion (3 weeks p.c.) in contrast to previous studies in goats [17]. This could be due to the high *M*. *caprae* dose used in the present study. Finally, in BCG vaccinated animals lower MPB83-IgG levels were observed, in concordance to previous studies in goats [8] and cattle [25].

HIMB induced similar cell-mediated and humoral immune responses in 7 out of 8 animals irrespectively of the parenteral vaccine delivery route (s.c. or i.m.). Intriguingly, one animal of the HIMB group (s.c.) did not show neither humoral nor cell-mediated responses after vaccination. Since this animal was only 4 weeks old at the vaccination point (2–3 weeks younger than the rest of experimental animals), this lack of response might be explained by a poorer capability of the immunological system of this animal to respond properly to the vaccination. This suggests that age of vaccination is an important variable that should be taken into consideration in future studies with HIMB vaccine.

Finally, at the end of the study, some control goats became less responsive or unresponsive to tuberculins, being negative to qualitative diagnose, which might suggest an exhaustion of cell-mediated specific response. Unresponsiveness due to exhaustion of cell mediated response in controls had already been observed in goat trials with tuberculosis, although unresponsiveness was observed at week 14 p.c. and challenge was performed with 10^3^ of *M*. *caprae* [17].

In conclusion, the results provide evidence that parenteral vaccination of goats with HIMB can be as protective against TB infection as BCG vaccination. Moreover, since it is an inactivated vaccine, HIMB is more stable and environmentally safer under field conditions than live attenuated BCG. Thus, HIMB vaccine may be an improved tool for goat TB vaccination programs. Further studies are required using other experimental conditions (namely, lower bacterial dose for challenge, different administration routes with larger number of animals, longer follow-up) and interaction with other environmental factors in field trials.

## Supporting information

S1 Table Raw data OD 450 nm of IFN-γ after vaccination and challenge. (XLSX)

S2 Table Raw data OD 450 nm of MPB83-IgG after vaccination and challenge. (XLSX)
